# RSL1D1 promotes the progression of colorectal cancer through RAN-mediated autophagy suppression

**DOI:** 10.1038/s41419-021-04492-z

**Published:** 2022-01-10

**Authors:** Xunhua Liu, Jianxiong Chen, Xiaoli Long, Jiawen Lan, Xiaoting Liu, Miao Zhou, Sijing Zhang, Jun Zhou

**Affiliations:** 1 Department of Pathology, Nanfang Hospital, Southern Medical University, Guangzhou, 510515 China; 2 Department of Pathology, School of Basic Medical Sciences, Southern Medical University, Guangzhou, 510515 China

**Keywords:** Colorectal cancer, Metastasis

## Abstract

RSL1D1 (ribosomal L1 domain containing 1), a member of the universal ribosomal protein uL1 family, was suggested to be a new candidate target for colorectal cancer (CRC). However, the role of RSL1D1 in cancer, including CRC, remains largely elusive. Here, we demonstrated that RSL1D1 expression was significantly elevated in tumors from CRC patients and that high expression of RSL1D1 was correlated with poorer survival of CRC patients. Functionally, RSL1D1 promoted the proliferation, invasion, and metastasis of CRC cells by suppressing autophagy. Interestingly, RSL1D1 interacted with RAN and inhibited its deacetylation by competitively binding with Sirt7. By affecting the acetylation of RAN, RSL1D1 inhibited the accumulation of nuclear STAT3 and the STAT3-regulated autophagic program. Taken together, our study uncovered the key role of the RSL1D1/RAN/STAT3 regulatory axis in autophagy and tumor progression in CRC, providing a new candidate target for CRC treatment.

## Introduction

Colorectal cancer (CRC) is a type of gastrointestinal cancer with high morbidity and mortality [1]. Although targeted therapy and immunotherapy have greatly improved the CRC treatments in recent years [2–4], tumor recurrence and metastasis represent the main causes of cancer-related death. Therefore, it is critical to identify additional key targets in CRC progression and metastasis.

RSL1D1, which contains part of the ribosomal L1p/L10e consensus sequence in its N-terminus and a long lysine-rich domain in its C-terminus, was suggested as a candidate target for CRC [5]. RSL1D1 participates in ribosome biosynthesis or acts as a transcriptional cofactor [6, 7]. In addition, RSL1D1, also named cellular senescence-inhibited gene (CSIG), is abundantly expressed in young human fibroblast cells, but its expression declines during cellular senescence, which can delay the process of replicative senescence by regulating the cell cycle [8]. Cellular senescence, a state of cell cycle arrest in aged cells, has been widely recognized as a potent tumor-suppressive mechanism [9]. As an inhibitor of cellular senescence, RSL1D1 is highly expressed in liver, prostate, and breast cancers, suggesting that it may be closely related to the pathogenesis and progression of many cancers [10–12]. Nevertheless, the role of RSL1D1 in CRC is still unclear.

Autophagy is a cellular degradation and recycling process that occurs in response to a variety of stress stimuli [13, 14]. A growing number of studies have suggested that autophagy participates in tumorigenesis, metastasis, and tumor therapy. Nevertheless, the roles of autophagy in different stages of cancer development might be controversial, depending on the specific context involved [15]. Moreover, as an important cellular stress reaction, autophagy possesses properties similar to those of cellular senescence. Accumulating reports have demonstrated that there is a strong relationship between autophagy and cellular senescence [16–18]. However, evidence for the role of RSL1D1 in the autophagic program is still lacking.

In this study, we found that the expression of RSL1D1 was upregulated in CRC and was correlated with poor prognosis. Notably, RSL1D1 suppresses the deacetylation of RAN by competitively interacting with Sirt7, which inhibits the accumulation of nuclear STAT3 and the autophagic program and then promotes the proliferation, invasion, and metastasis of CRC. Our findings provide a deeper understanding of RSL1D1 during CRC progression and may contribute to the development of new therapeutic targets for CRC.

## Results

### RSL1D1 is highly expressed in tumor samples from CRC patients, and high expression of RSL1D1 is correlated with poor CRC prognosis

**Fig. 1:**
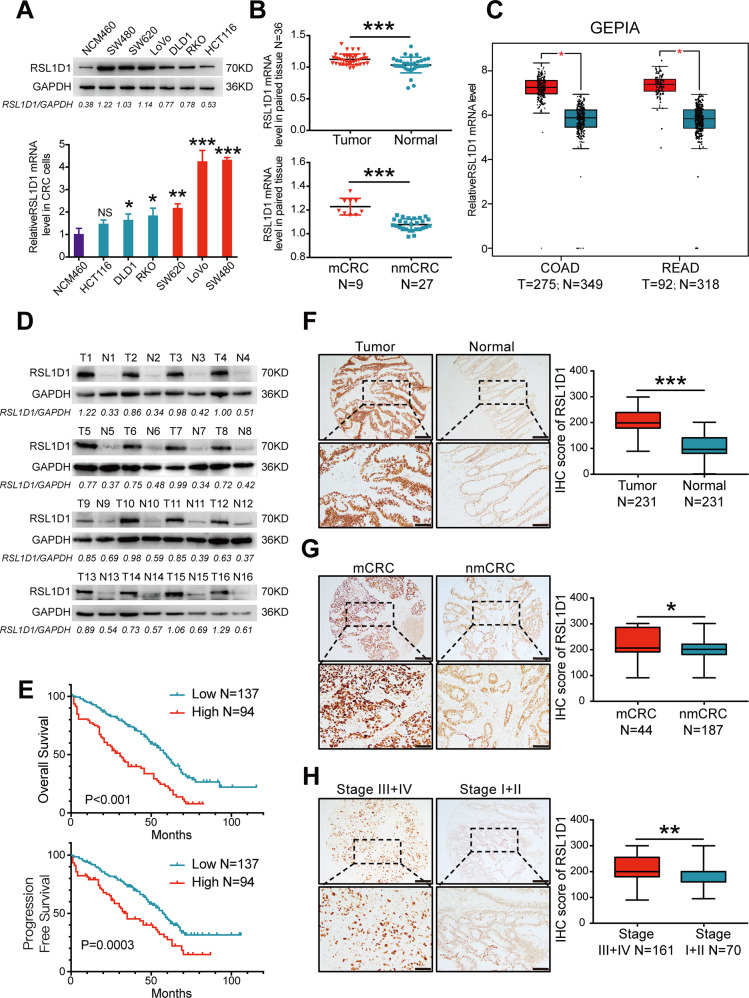
RSL1D1 is highly expressed in tumor samples from patients with CRC, and high expression of RSL1D1 is correlated with poor CRC prognosis. **A** Total mRNAs and proteins were extracted from 6 CRC cell lines and 1 normal intestinal epithelial cell line by RT-PCR and WB, respectively. **B** qRT-PCR analysis of the relative RSL1D1 mRNA expression in 36 paired CRC tissue samples. [mCRC]: CRC with distant metastasis; [nmCRC]: CRC without distant metastasis. **C** The RSL1D1 mRNA expression of CRC samples from TCGA was analyzed by Gene Expression Profiling Interactive Analysis (GEPIA). [COAD]: Colon adenocarcinoma, [READ]: rectal adenocarcinoma. The red * means *P* < 0.01. **D** WB analysis of the RSL1D1 protein in 16 cases of CRC tumors. [T]: Colorectal cancer tumors, [N]: matched adjacent colonic mucosa. **E** Kaplan–Meier curve depicting the survival time of patients with CRC (*N* = 231). The curves were stratified based on the RSL1D1 level and scored by intensity (0~3) and area (0~100) from IHC technology. **F–H**. Scores for RSL1D1 staining in the TMA from 231 patients with CRC by IHC staining. Representative images of IHC staining for RSL1D1 are shown; magnification scale bar, 100 µm; scale bar in the enlarged image, 50 µm. The right panels show the corresponding statistical results. **[F]**. IHC staining score of 231 CRC cases and paired adjacent normal mucosa; **[G]***.* IHC staining score of non-metastatic and metastatic paraffin-embedded CRC tissues of 231 cases. **[H]***.* IHC staining score of CRC cases respectively belonging to Stage I + II and Stage III + IV. *P < 0.05, **P < 0.01, ***P < 0.001, NS means no statistic difference, except the * of Fig. 1C. The error bars represent mean ± SD.

The mRNA and protein expression levels of RSL1D1 were studied in 6 CRC cell lines (SW480, SW620, LoVo, DLD1, HCT116, and RKO), 1 intestinal epithelial cell line (NCM460) and paired CRC tissues with normal counterparts using quantitative reverse transcription-polymerase chain reaction (qRT-PCR) and Western blot (WB) assays. At both the mRNA and protein levels, RSL1D1 was highly expressed in CRC cell lines compared with that in the normal epithelial cell line NCM460 (Fig. 1A). In clinical samples, RSL1D1 expression was significantly increased in primary CRC tissue samples compared with their normal counterparts at the mRNA (*N* = 36, *P* < 0.001, Fig. 1B) and protein levels (*N* = 16, Fig. 1D). In addition, the expression of RSL1D1 mRNA in primary tumors from CRC patients with distant metastasis was higher than that in primary tumors from CRC patients without distant metastasis (*P* < 0.001, Fig. 1B). We further analyzed the mRNA expression of RSL1D1 in CRC samples using GEPIA public data [19], and the results showed that the expression of RSL1D1 mRNA in CRC tumor tissues was higher than that in normal intestinal tissues (*P* < 0.01, Fig. 1C).

Tumor samples from 231 CRC patients were used to analyze the correlation between RSL1D1 expression and the clinicopathological characteristics or prognosis of CRC patients by immunohistochemical (IHC) staining. IHC staining for RSL1D1 protein expression was scored by multiplying the intensity value (0–3) and percentage of the staining area (0–100). Our results showed that RSL1D1 expression in tumors from CRC patients was significantly higher than that in adjacent normal mucosa (*P* < 0.001, Fig. 1F). In addition, RSL1D1 expression was greatly increased in primary tumors from CRC patients with distant metastases (*P* = 0.028, Fig. 1G), node metastases (*P* = 0.005, Supplementary Table 1) and an advanced clinical stage (*P* = 0.004, Fig. 1H). Notably, the Kaplan–Meier survival test showed that CRC patients with high RSL1D1 expression had poorer long-term survival (overall survival: LogRank=27.28, *P* < 0.001; progression-free survival: LogRank=12.78, *P* = 0.0003, Fig. 1E).

In general, our results indicated that RSL1D1 was highly expressed in tumor samples from patients with CRC and that high expression of RSL1D1 was correlated with poor prognosis.

### Overexpression of RSL1D1 promotes CRC cell proliferation, invasion, and metastasis in vitro and in vivo

**Fig. 2:**
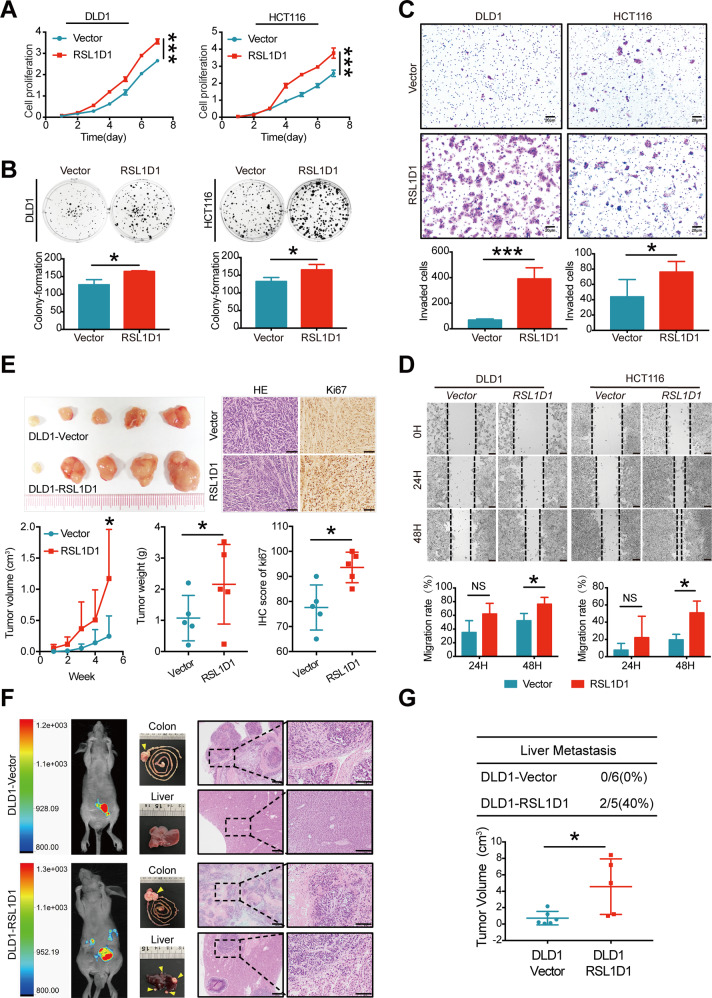
Overexpression of RSL1D1 promotes CRC cell proliferation, invasion, and metastasis in vitro and in vivo. **A, B** The proliferative ability of RSL1D1-overexpressing CRC cells was analyzed by CCK-8 assay [**A**] and plate colony formation assay [**B**]. **C** Transwell invasion assays assessed the invasive ability of RSL1D1-overexpressing CRC cells. Scale bar, 20 µm. **D** A wound-healing assay assessed the migration capability of RSL1D1-overexpressing CRC cells. Scale bar, 50 µm. **E** Photographs of the subcutaneous tumors of nude mice constructed with RSL1D1-overexpressing CRC cells and control cells. Tumor growth was measured by volume (cm^3^) and weight (g). H&E and Ki-67 staining of subcutaneous tumors are shown. Scale bar, 50 µm. **F** Representative photographs of nude mice after cecum injection with RSL1D1-overexpressing CRC cells and control cells: whole-body fluorescence optical imaging 45 days postinoculation (left), macroscopic pictures of the primary cancer in the colon and the liver (middle), and micrographs of the organs shown by H&E staining (right). Magnification scale bar 100 µm; scale bar in enlarged image, 50 µm. **G** Summary of the results of the nude mice presenting with liver metastasis between RSL1D1-overexpressing CRC cells and control cells. The primary tumor growth rate was measured by volume (cm^3^). *P < 0.05, **P < 0.01, ***P < 0.001, NS means no statistic difference. The error bars represent mean ± SD.

To further investigate the function of RSL1D1 in CRC, we overexpressed RSL1D1 in DLD1 and HCT116 cells by lentivirus transfection (DLD1-Vector/DLD1-RSL1D1, HCT116-Vector/HCT116-RSL1D1). WB and qRT-PCR analyses were used to test transfection effectiveness (Fig. S1A). CCK-8 assay and colony formation assay showed that upregulated RSL1D1 significantly promoted cellular proliferation (Fig. 2A) and colony formation of CRC cells (Fig. 2B). Moreover, Matrigel invasion and wound healing assays indicated that RSL1D1 overexpression significantly promoted the invasion and migration of CRC cells at 48 h (Fig. 2C, D).

To study the effects of RSL1D1 overexpression on the tumor growth and metastasis of CRC cells in vivo, mouse models of subcutaneous xenografted tumor growth and liver metastasis were generated. To establish an orthotopic liver metastasis model in nude mice, we injected CRC cells into the subserosa of the cecum. As indicated, RSL1D1 overexpression significantly promoted DLD1 cell growth in vivo (Fig. 2E). In addition, IHC staining confirmed that tumors with RSL1D1 overexpression (DLD1-RSL1D1 cells) showed a higher Ki-67 proliferation index than control tumors (DLD1-Vector cells) (*P* = 0.02, Fig. 2E). For the liver metastasis model, 45 days after implantation, we found that more metastatic nodules in the liver and larger colon tumors developed in mice implanted with RSL1D1-overexpressing CRC cells than in mice implanted with control CRC cells (Fig. 2F, G).

Collectively, our results demonstrated that RSL1D1 overexpression promoted the growth and metastasis of CRC cells in vitro and in vivo, strongly indicating that RSL1D1 functions as a tumor promoter in CRC.

### Knockdown of RSL1D1 inhibits CRC cell proliferation, invasion, and metastasis in vitro and in vivo

To further test the functions of RSL1D1 in CRC cells, we established two CRC cell lines with stable RSL1D1 knockdown using a lentiviral shRNA technique (SW480-CTRL/SW480-shRSL1D1, LoVo-CTRL/LoVo-shRSL1D1). The knockdown efficiencies were confirmed by WB and qRT-PCR (Fig. S1B).

**Fig. 3:**
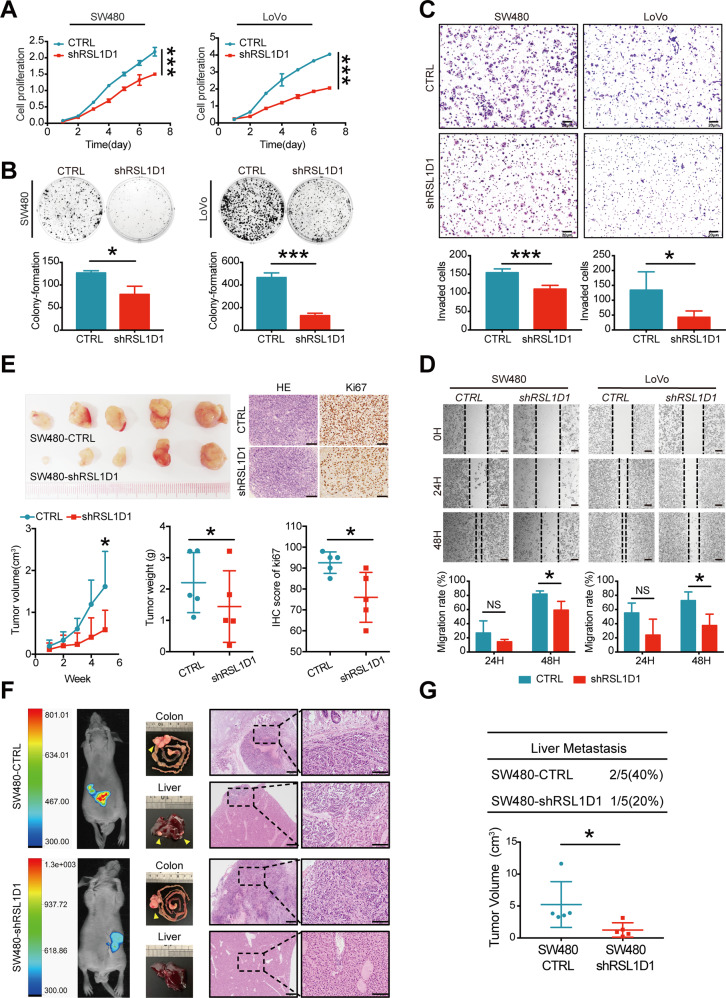
Knockdown of RSL1D1 inhibits CRC cell proliferation, invasion, and metastasis in vitro and in vivo. **A, B** The proliferative ability of RSL1D1-downregulated CRC cells was analyzed by CCK-8 assay [**A**] and plate colony formation assay [**B**]. **C** Transwell invasion assays assessed the invasive ability of RSL1D1-downregulated CRC cells. Scale bar, 20 µm. **D** A wound healing assay assessed the migration capability of RSL1D1-downregulated CRC cells. Scale bar, 50 µm. **E** Photographs of the subcutaneous tumors of nude mice constructed with RSL1D1-downregulated CRC cells and control cells. Tumor growth was measured by volume (cm^3^) and weight (g). H&E and Ki-67 staining of subcutaneous tumors are shown. Scale bar, 50 µm. **F** Representative photographs of nude mice after cecum injection with RSL1D1-downregulated CRC cells and control cells: whole-body fluorescence optical imaging 45 days postinoculation (left), macroscopic pictures of the primary cancer in the colon and the liver (middle), and micrographs of the organs shown by H&E staining (right). Magnification scale bar 100 µm; scale bar in enlarged image, 50 µm. **G** Summary of the results of the nude mice presenting with liver metastasis between RSL1D1-downregulated CRC cells and control cells. The primary tumor growth rate was measured by volume (cm^3^). * *P* < 0.05, ** *P* < 0.01, ***P < 0.001, NS means no statistic difference. The error bars represent mean ± SD.

As shown in Fig. 3A–D, knockdown of RSL1D1 inhibited the proliferation, migration, and invasion of SW480 and LoVo cells in vitro. The subcutaneous tumor model showed that knockdown of RSL1D1 inhibited the tumor growth of SW480 cells in vivo (Fig. 3E). The orthotopic liver metastasis model of mice demonstrated that knockdown of RSL1D1 suppressed liver metastasis of SW480 cells in vivo (Fig. 3F, G).

In summary, our results demonstrated that RSL1D1 knockdown inhibited the growth and metastasis of CRC cells in vitro and in vivo.

### RSL1D1 promotes CRC cell proliferation and invasion by suppressing autophagy

**Fig. 4:**
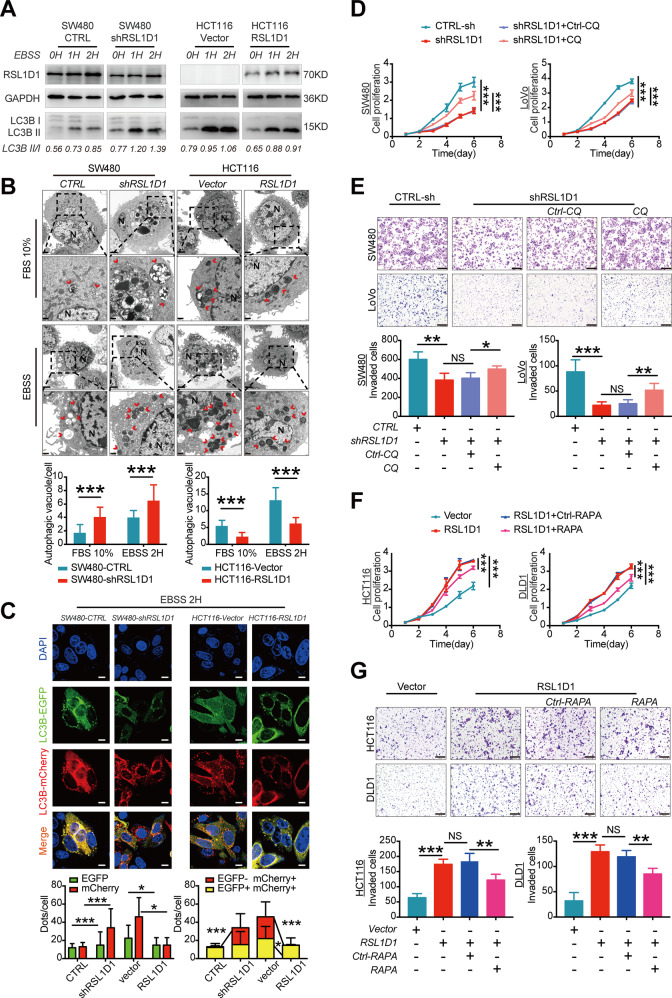
RSL1D1 promotes CRC cell proliferation and invasion by suppressing autophagy. **A** Protein expression of LC3B was examined by WB when RSL1D1 was downregulated and upregulated in CRC cells under starvation in Earle’s balanced salt solution (EBSS). **B** Electron microscopy images presenting the ultrastructure of the CRC cells after RSL1D1 knockdown and overexpression under normal conditions or starvation conditions. Red arrows indicate autophagic vacuoles (AVs). The number of AVs in 10 cells was counted in each section. Magnification scale bar, 2 µm; scale bar in the enlarged image, 500 nm. **C** CRC cells with RSL1D1 downregulation and overexpression were transfected with mCherry-EGFP-LC3B, and the changes in green and red fluorescence were observed using a confocal microscope under starvation conditions. Scale bar, 20 µm. The numbers of green and red dots per cell in each condition were quantified with 20 cells counted in each group. **D**–**E** CCK-8 [**D**] and Transwell invasion assays [**E**] were used to detect the proliferation and invasion of CRC cells after RSL1D1 knockdown or CQ (10 µM) treatment for 24 h. Scale bar, 100 µm. **F**–**G** CCK-8 [**F**] and Transwell invasion assays [**G**] were used to detect the proliferation and invasion of CRC cells after RSL1D1 overexpression or RAPA (100 nM) treatment for 24 h. Scale bar, 100 µm. *P < 0.05, **P < 0.01, ***P < 0.001, NS means no statistic difference. The error bars represent mean ± SD.

RSL1D1, also named cellular senescence-inhibited gene (CSIG), plays a critical role in cellular senescence [8, 20]. There are some functional connections between autophagy and cellular senescence [16]. Therefore, we speculated that RSL1D1 may participate in the autophagic program by which RSL1D1 promotes CRC cell proliferation, invasion, and metastasis. Based on this assumption, public data of CRC were obtained from The Cancer Genome Atlas (TCGA), and Gene Ontology (GO) was performed by using R DESeq2 and the clusterprofiler package [21]. Interestingly, RSL1D1 was suggested to be involved in the autophagic program of CRC (Fig. S2A). Then, we examined the induction of autophagy by WB analysis of the ratio of LC3B II/I and P62 [22]. Interestingly, increased LC3B II/I and decreased P62 expressions were observed in SW480 and LoVo cells after RSL1D1 knockdown. Moreover, RSL1D1 overexpression suppressed autophagy in DLD1 and HCT116 cells (Fig. S2B), indicating as decreased LC3B II/I and increased P62 expressions. Given that starvation initiates the autophagic program, we further evaluated the effects of RSL1D1 on the starvation-mediated autophagic program. Cells were cultured under starvation in Earle’s balanced salt solution (EBSS) for different intervals, and we found that RSL1D1 knockdown aggravated the starvation-initiated autophagic program, while RSL1D1 overexpression attenuated the starvation-initiated autophagic program of CRC cells (Fig. 4A). Transmission electron microscopy was employed to observe the ultrastructure of the cells. Our results revealed that RSL1D1 knockdown significantly increased the number of autophagic vacuoles (AVs) under normal conditions or starvation conditions, while RSL1D1 overexpression reduced the number of AVs under normal conditions or starvation conditions (Fig. 4B). To further quantify the changes in autophagy levels, the mCherry-EGFP-LC3 reporter system was used to analyze the changes in autophagic flux after RSL1D1 knockdown or overexpression. Confocal images showed that the numbers of both EGFP (green) and mCherry (red) dots were significantly increased in CRC cells after RSL1D1 knockdown under both normal and starvation conditions (Figs. 4C, S2C). In the merged images, more free red dots were seen than yellow dots, indicating significantly increased autolysosome formation compared with autophagosomes, suggesting that autophagic flux was increased in CRC cells with RSL1D1 knockdown. In addition, decreased autophagic flux was found in CRC cells after RSL1D1 overexpression under both normal and starvation conditions (Figs. 4C, S2C).

To explore the functional role of the RSL1D1-regulated autophagic program in the proliferation or invasion of CRC cells, we treated CRC cells with the autophagy inhibitor chloroquine (CQ; final concentration: 10 µM) for 24 h after RSL1D1 knockdown (Fig. S2D). Interestingly, RSL1D1 knockdown-mediated inhibitory proliferation and invasion of CRC cells were successfully rescued by CQ treatment (Fig. 4D, E). Moreover, rapamycin (RAPA; final concentration: 100 nM), a classical autophagy stimulator, was used in CRC cells after RSL1D1 overexpression for 24 h (Fig. S2E). The promotion of CRC cell proliferation and invasion after RSL1D1 overexpression was inhibited by RAPA treatment (Fig. 4F, G).

In brief, RSL1D1 promoted CRC cell proliferation and invasion by suppressing autophagy.

### The interaction between RSL1D1 and RAN is essential for autophagy and the proliferation and invasion of CRC cells

**Fig. 5:**
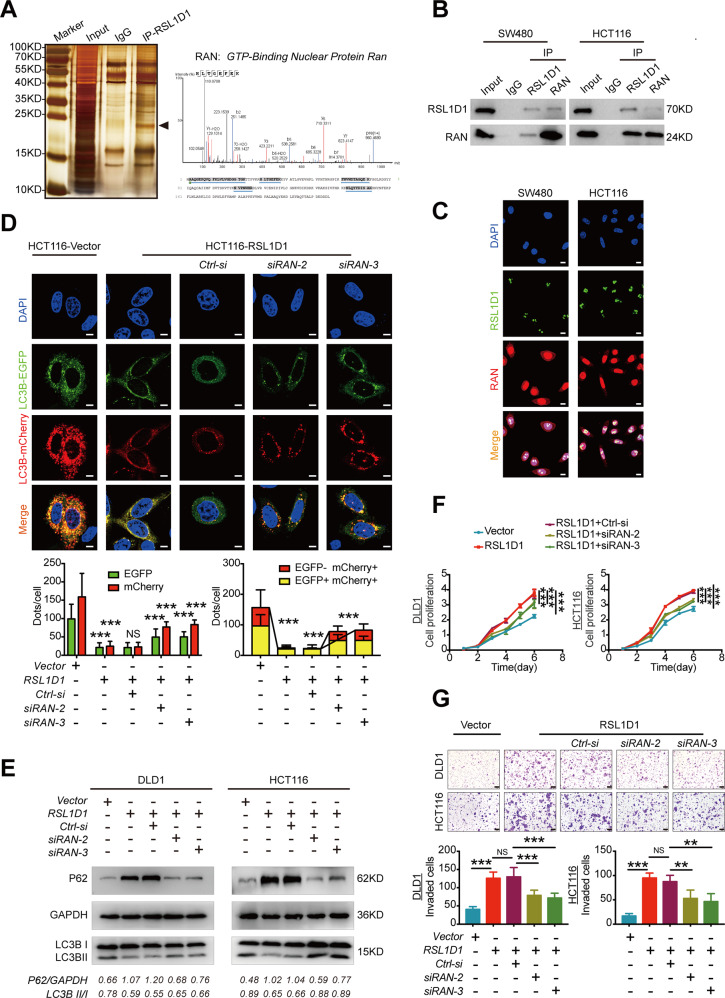
The interaction between RSL1D1 and RAN is essential for autophagy and the proliferation and invasion of CRC cells. **A** Co-IP combined with LC-MS/MS analysis was used to analyze the interacting proteins of RSL1D1 in CRC cells; RAN, a ras-related nuclear protein, was identified. **B** Co-IP of endogenous RSL1D1 and RAN proteins in CRC cells. **C** Double IF staining revealed the colocalization of RSL1D1 and RAN proteins in CRC cells. Scale bar, 10 µm. **D** The autophagic flux of CRC cells with stable RSL1D1 knockdown and transient RAN knockdown under starvation conditions was monitored by the mCherry-EGFP-LC3B assay. Scale bar, 50 µm. **E** Protein expression of LC3-II and P62 was examined by WB in CRC cells with stable RSL1D1 knockdown and transient RAN knockdown under starvation conditions. **F**–**G**. CCK-8 [**F**] and Transwell invasion assays [**G**] were used to detect the proliferation and invasion of CRC cells after stable RSL1D1 knockdown and transient RAN knockdown. Scale bar, 50 µm. *P < 0.05, **P < 0.01, ***P < 0.001, NS means no statistic difference. The error bars represent mean ± SD.

To further explore the mechanism by which RSL1D1 inhibits autophagy in CRC cells, we applied coimmunoprecipitation (Co-IP) combined with liquid chromatography-tandem mass spectrometry (LC-MS/MS) to identify candidates for RSL1D1-interacting proteins. RAN, a member of the RAS superfamily of small GTPases, was identified as one of the RSL1D1-interacting protein candidates (Fig. 5A). Interestingly, the GO analysis [21] also predicted that RSL1D1 can bind with RAS GTPases (Fig. S2A). Co-IP assays showed that RSL1D1 could bind with RAN in CRC cells endogenously (Fig. 5B). Immunofluorescence (IF) localization by confocal microscopy revealed that RSL1D1 and RAN were colocalized mainly in the nuclei of CRC cells (Fig. 5C).

To establish the functional role of the interaction between RSL1D1 and RAN in the autophagic program and biological behavior of CRC cells, we used siRNAs targeting RAN to knockdown RAN expression in CRC cells after RSL1D1 overexpression and observed the effects of RAN knockdown on autophagy, proliferation, and invasion. The efficiency of RAN knockdown was confirmed by WB and qRT-PCR (Fig. S1C). Importantly, the inhibitory effects of autophagy in CRC cells after RSL1D1 overexpression under starvation conditions were successfully abolished by RAN knockdown (Fig. 5D, E). Furthermore, RSL1D1 overexpression-mediated enhanced proliferation and invasion of CRC cells were attenuated by RAN downregulation (Fig. 5F, G).

Together, these data demonstrated that RAN plays a critical role in the process by which RSL1D1 suppresses autophagy and promotes the proliferation and invasion of CRC cells.

### The interaction between RSL1D1 and RAN helps to influence the accumulation of nuclear STAT3 and the STAT3-regulated autophagy program

**Fig. 6:**
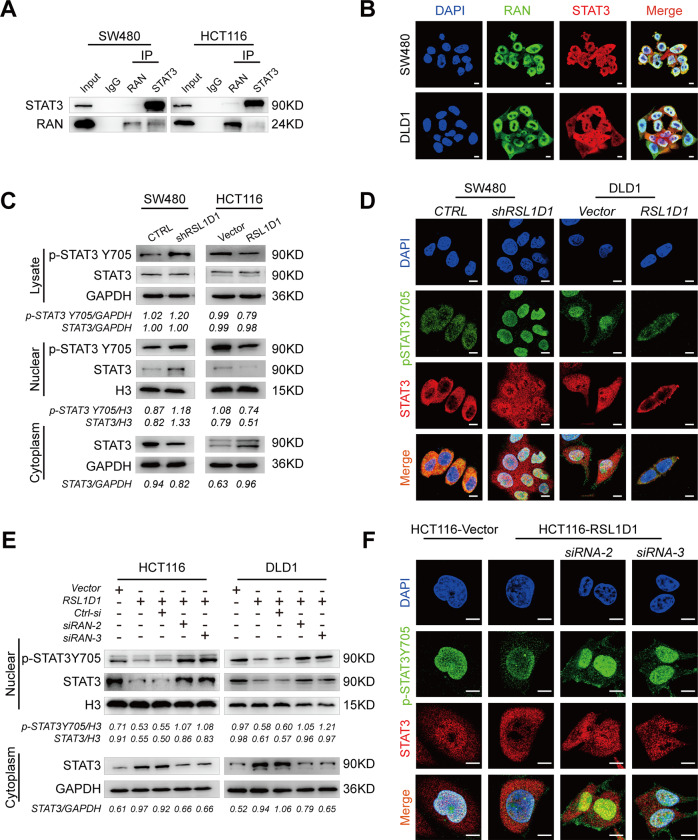
The interaction between RSL1D1 and RAN helps to inhibit the accumulation of nuclear STAT3 and the STAT3-regulated autophagy program. **A** Co-IP of endogenous RAN and STAT3 proteins in CRC cells. **B** Double IF staining revealed the colocalization of RAN and STAT3 proteins in CRC cells. Scale bar, 10 µm. **C** Total/Nuclear/cytoplasmic extraction and western blotting detected the expression of STAT3 and p-STAT3Y705 in CRC cells after RSL1D1 knockdown and overexpression. **D** IF analysis of endogenous STAT3 and p-STAT3Y705 in CRC cells after RSL1D1 knockdown and overexpression. Scale bar, 20 µm. **E**. Nuclear/cytoplasmic extraction and western blotting detected the expression of STAT3 and p-STAT3 in CRC cells after RSL1D1 overexpression and RAN transient knockdown under starvation conditions. **F** Double IF staining of p-STAT3Y705 and STAT3 proteins in HCT116 cells after RSL1D1 overexpression and RAN transient knockdown under starvation. Scale bar, 50 µm.

Autophagy is commonly seen as a cytoplasmic event. However, recent studies have unveiled a transcriptional and epigenetic network that regulates autophagy [23]. It was suggested that signal transducer and activator of transcription-3 (STAT3) nuclear import was dependent on RAN and STAT3 plays a key role in autophagy due to its different subcellular localization; [24, 25] therefore, we speculated that the interaction between RSL1D1 and RAN may participate in the autophagic program by affecting STAT3 subcellular distribution. Accordingly, an interaction between RAN and STAT3 was detected by Co-IP (Fig. 6A), and IF assays further confirmed the colocalization of RAN and STAT3 in CRC cells (Fig. 6B). To further study the effects of RSL1D1 on the STAT3 protein, total/nuclear/cytoplasmic STAT3 and p-STAT3Y705 in CRC cells were analyzed. Interestingly, WB and IF assays indicated that RSL1D1 knockdown led to increased p-STAT3Y705 and nuclear STAT3 but decreased cytoplasmic STAT3 in CRC cells. Accordingly, RSL1D1 overexpression resulted in decreased p-STAT3Y705 and nuclear STAT3, while increasing cytoplasmic STAT3 in CRC cells (Fig. 6C, D). In addition, there were no effects on the total expression level of STAT3 in CRC after RSL1D1 knockdown and overexpression, suggesting that RSL1D1 actually affects the subcellular distribution of STAT3 (Fig. 6C). qRT-PCR analysis showed that the mRNA levels of STAT3-regulated autophagy genes (BINP3, BCL-2, BECN1, ATG3, ULK1, ATG5, LC3B) were significantly elevated when RSL1D1 expression was knocked down in SW480 cells (Fig. S3A). To further investigate the effects of the interaction between RSL1D1 and RAN on STAT3 distribution, we knocked down RAN in CRC cells after RSL1D1 overexpression. WB and IF assays demonstrated that RSL1D1 overexpression-mediated decreases in the nuclear STAT3 and p-STAT3Y705 levels were greatly reversed by RAN knockdown (Fig. 6E, F). To exclude the possibility that RSL1D1 affects STAT3 distribution in addition to RAN, we also detected the endogenous interaction between RSL1D1 and the STAT3 protein in CRC cells, and the Co-IP assay showed that they could not coprecipitate (Fig. S3B).

Collectively, the interaction between RSL1D1 and RAN influences the accumulation of nuclear STAT3 and the STAT3-regulated autophagy program.

### RSL1D1 inhibits the deacetylation of RAN by competitively binding with Sirt7 and then promotes CRC cell proliferation and invasion by suppressing autophagy

**Fig. 7:**
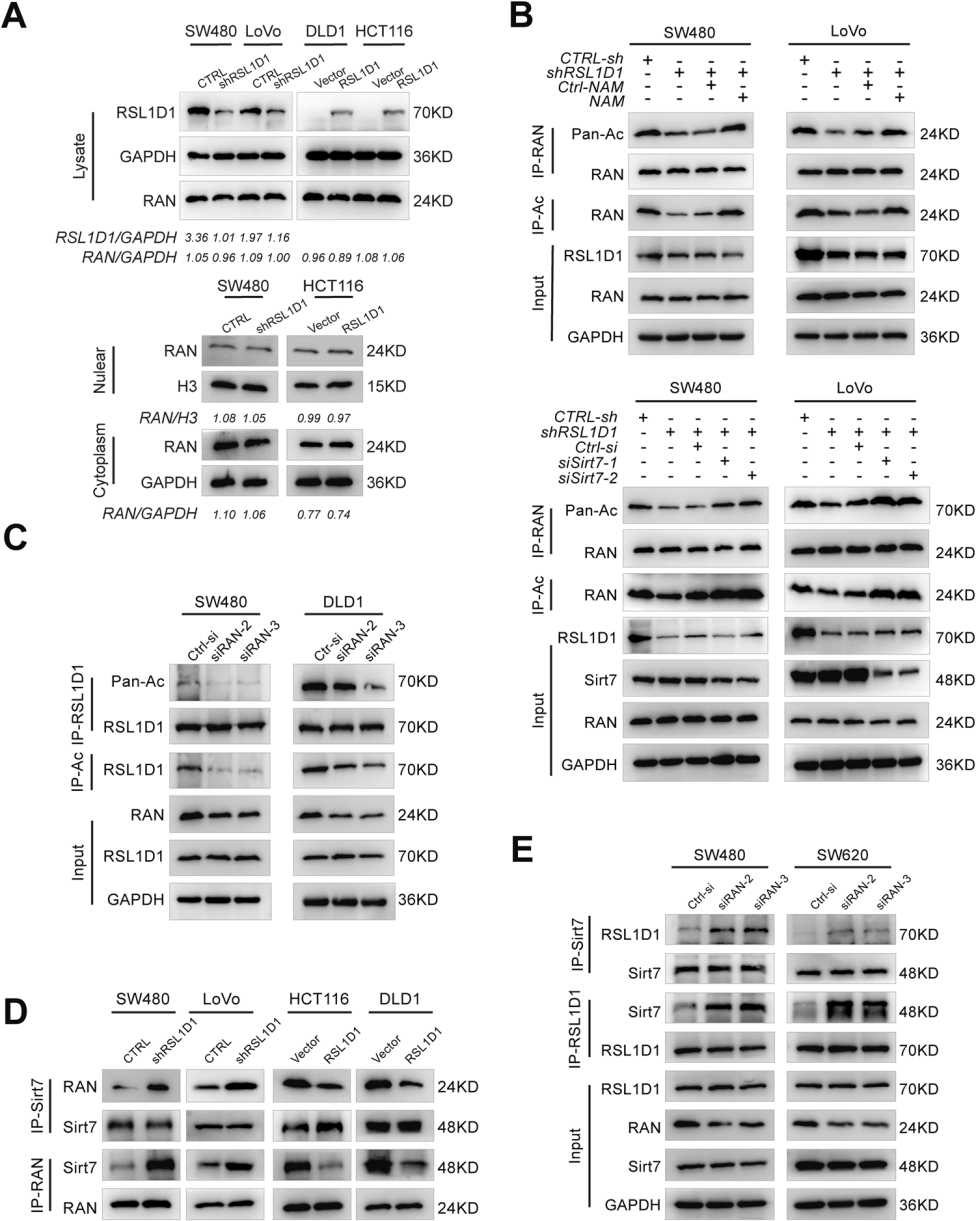
RSL1D1 inhibits the deacetylation of RAN by competitively binding with Sirt7. **A** Total/nuclear/cytoplasmic extraction and western blot analysis of RAN expression in CRC cells after RSL1D1 knockdown and overexpression. **B** Acetylation of RAN in CRC cells after RSL1D1 knockdown and treatment with the deacetylase inhibitor NAM or transfection with siRNA-Sirt7. **C** Acetylation of RSL1D1 in CRC cells transfected with siRNA-RAN. **D** Co-IP of the interaction between RAN and Sirt7 in CRC cells after RSL1D1 knockdown or overexpression. **E** Co-IP of the interaction between RSL1D1 and Sirt7 in CRC cells after transient RAN knockdown.

Although our previous data confirmed that there was a protein interaction between RSL1D1 and RAN, the exact mechanism remains unclear. As we known, the protein expression level is critical for their function, we then detected whether the expression level of the RAN protein was regulated by RSL1D1 in CRC cells. However, our results showed that RSL1D1 overexpression or knockdown had no effect on the expression levels of the RAN protein in CRC cells (Fig. 7A). Considering that RAN is an essential transport protein [26], we further examined the potential effect of RSL1D1 on the nucleocytoplasmic distribution of RAN protein, the results showed that there was no significant difference in the nucleocytoplasmic distribution of RAN in CRC cells after RSL1D1 overexpression or knockdown (Fig. 7A)

Recent studies have found that RAN is regulated by lysine acetylation and deacetylated by Sirt7; [27, 28] therefore, we speculated that acetylation of RAN, which is essential for the different subcellular localizations of STAT3 caused by RSL1D1, may be related to the interaction between RAN and Sirt7. We then examined the acetylation levels of endogenous RAN in CRC cells treated with nicotinamide (NAM), an inhibitor of Sirt family deacetylases, or siRNAs targeting Sirt7. Using a specific antibody against acetylated lysine, we detected strong acetylation levels of RAN in NAM-treated cells or Sirt7 knockdown cells (Fig. S4E). Moreover, Co-IP assays and IF staining showed that Sirt7 interacted with RAN (Fig. S4A) and was colocalized with RAN in the nuclei of CRC cells (Fig. S4B). Interestingly, we found that RAN acetylation was significantly decreased in CRC cells after RSL1D1 knockdown, while the acetylation levels of RAN were greatly increased in CRC cells overexpressing RSL1D1 (Fig. S4G). More importantly, inhibition of Sirt7 function by NAM or siRNA-Sirt7 greatly rescued the RSL1D1 knockdown-mediated reduction in acetylation levels of RAN in CRC cells (Fig. 7B).

**Fig. 8:**
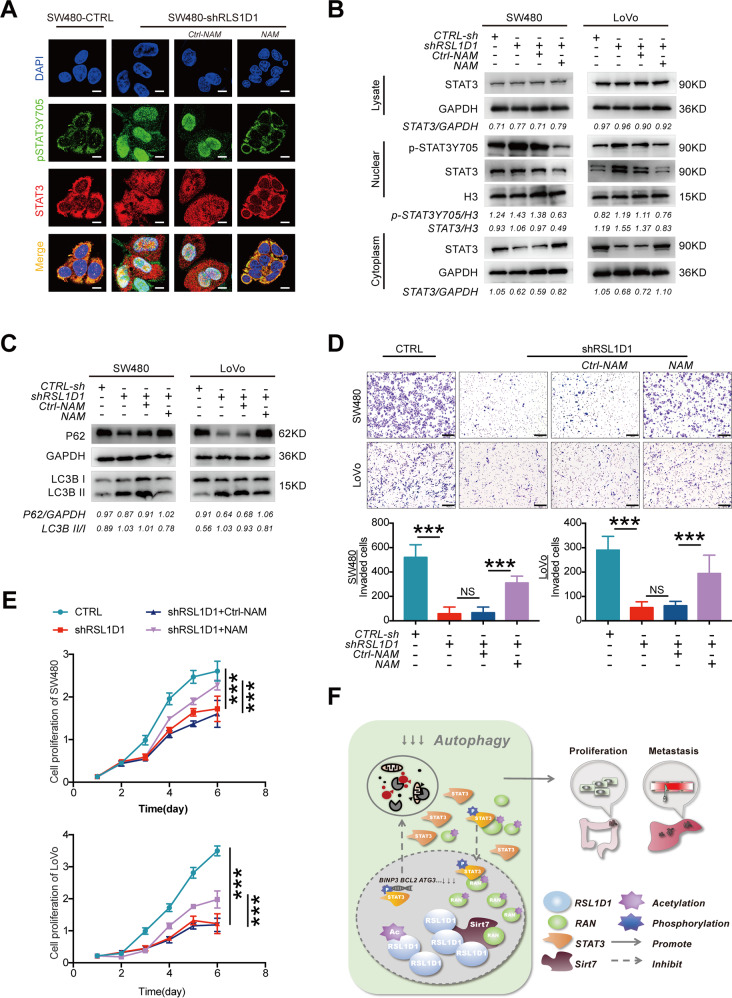
RSL1D1 inhibits the deacetylation of RAN and then promotes CRC cell proliferation and invasion by suppressing STAT3-regulated autophagy. **A** IF analysis of STAT3 and p-STAT3 in CRC cells after RSL1D1 knockdown treated with NAM. Scale bar, 20 µm. **B** Total/nuclear/cytoplasmic extraction and western blotting detected the expression of STAT3 and p-STAT3Y705 in CRC cells after RSL1D1 knockdown and treatment with NAM. **C** Protein expression of LC3-II and P62 was examined by WB in CRC cells after RSL1D1 knockdown and treatment with NAM. **D**–**E**. CCK-8 [**D**] and Transwell invasion assays [**E**] were used to detect the proliferation and invasion of CRC cells after RSL1D1 knockdown and treatment with NAM. Scale bar, 100 µm. **F** Schematic representation of the function and potential mechanism of RSL1D1 in colorectal cancer. *P < 0.05, **P < 0.01, ***P < 0.001, NS means no statistic difference. The error bars represent mean ± SD.

From proteomics research, RSL1D1 was predicted to be an acetylated protein that interacts with Sirt7 [29]. To verify this, we tested the acetylation levels of endogenous RSL1D1 and exogenously expressed RSL1D1 in CRC cells, and a Co-IP assay and IF staining were used to study the interaction between RSL1D1 and Sirt7 in CRC cells and 293 T cells. The results showed that RSL1D1 was an acetylated protein and could be specifically deacetylated by Sirt7 (Fig. S4C, D, F). More interestingly, we found that silencing RAN reduced the acetylation of RSL1D1 in CRC cells (Fig. 7C). These results suggested that Sirt7 was responsible for the acetylated alteration between RSL1D1 and RAN. To further test this hypothesis, Co-IP assays were continually used for the semiquantitative analysis of the interactions among RSL1D1, RAN, and Sirt7. Our results showed that the interaction between RAN and Sirt7 was upregulated after RSL1D1 knockdown but downregulated after RSL1D1 overexpression in CRC cells (Fig. 7D). Similarly, the interaction between RSL1D1 and Sirt7 was upregulated after RAN knockdown in CRC cells (Fig. 7E). Furthermore, IF assays and WB showed that NAM treatment alleviated RSL1D1 knockdown-mediated increases in nuclear STAT3 and p-STAT3 in CRC cells after RSL1D1 knockdown (Fig. 8A, B). Accordingly, RSL1D1 knockdown increased autophagic flux, and this increase was abolished by NAM treatment (Fig. 8C). The inhibited proliferation and invasion of CRC cells after RSL1D1 knockdown were relieved by NAM treatment, as expected (Fig. 8D, E).

These findings suggested that RSL1D1 inhibited the deacetylation of RAN by competitively binding with Sirt7, which reduced the accumulation of nuclear STAT3 and then promoted the proliferation and invasion of CRC cells by suppressing autophagy.

## Discussion

Cellular senescence, a state of irreversible growth arrest, can be triggered by multiple mechanisms. Notably, dysregulation of cellular senescence is closely correlated with aberrant or excessive cellular proliferation, such as cancer [9, 30]. RSL1D1, also named cellular senescence-inhibited gene [8], was suggested to be a candidate target for CRC [5], but the role of RSL1D1 in CRC is still unclear.

Our study indicated that RSL1D1 might serve as a molecular marker for the prognosis and treatment of patients with CRC. We found that RSL1D1 was highly expressed in human CRC tissues and CRC cell lines, and higher RSL1D1 expression was correlated with poor prognosis. Functionally, RSL1D1 overexpression significantly promoted the proliferation and invasion of CRC cells in vitro and tumor growth and metastasis of CRC cells in vivo. Our findings suggested that RSL1D1 may be a tumor-promoting gene in CRC. Accordingly, several studies revealed that RSL1D1 could also promote the progression of other cancers, including liver carcinoma and prostate cancer [10, 12, 31].

Here, we also demonstrated that RSL1D1 promoted the progression of colorectal cancer by suppressing autophagy. RSL1D1 was previously found to play a critical role in cellular senescence [8, 20]. Furthermore, many stimuli that induce senescence also induce autophagy [16]. Interestingly, we found that RSL1D1 suppressed the autophagic program in CRC cells. Mechanistically, RSL1D1 promoted the progression of colorectal cancer by suppressing autophagy. Rapamycin (a classical autophagy stimulator) treatment greatly abolished the promotion of proliferation and invasion of CRC cells after RSL1D1 overexpression. In addition, treatment with the autophagy inhibitor chloroquine successfully alleviated the inhibitory proliferation and invasion of CRC cells mediated by RSL1D1 knockdown. These results implied that the autophagic program inhibited by RSL1D1 is a key mechanism involved in the tumor progression of CRC. The importance of autophagy in cancer was recently highlighted [15]. It is thought that autophagy plays dual roles in tumor. Autophagy suppresses tumorigenesis by reducing the accumulation of, or imbalance in, levels of some cellular components [32, 33], but autophagy also facilitates tumor progression by protecting the survival of some tumor cells in the nutrient-deprived tumor microenvironment [34, 35]. In summary, these studies showed that the role of autophagy in tumor progression depends on the context involved in.

Furthermore, we found that the interaction between RSL1D1 and RAN is essential for CRC cell autophagy and proliferation and invasion. We identified the protein RAN as an interacting protein of RSL1D1 in CRC cells, by which RSL1D1 transcriptionally suppressed autophagic gene expression by inhibiting the accumulation of nuclear STAT3. RAN, a member of the Ras superfamily of small GTPases, regulates nucleocytoplasmic transport, cell cycle progression, the nuclear envelope structure, and function [26]. Nuclear transport is one of the primary functions of the RAN system, and RAN dysfunction leads to abnormal nuclear trafficking of many proteins [36–38]. Interestingly, the nuclear import of STAT3 was found to be dependent on the function of RAN [24], and subcellular localization of STAT3 regulated by the nuclear import of STAT3 contributed to the autophagic program [25].

Another important finding herein was that RSL1D1 inhibited the deacetylation of RAN by competitively binding with Sirt7, which reduced the accumulation of nuclear STAT3 and then promoted the proliferation and invasion of CRC cells by suppressing autophagy. First, RAN is an acetylated protein and can be specifically deacetylated by Sirt7 [28, 39]. Sirt7 is a member of the sirtuin family and plays a critical role in cellular senescence [40, 41]. In our study, RSL1D1 promoted the acetylation level but not the protein expression level or subcellular localization of RAN, suggesting that RSL1D1 may participate in RAN posttranslational modification. Furthermore, we also found that RSL1D1 interacted with Sirt7 and was deacetylated by Sirt7, as predicted [29]. Notably, we found a competitive interaction with Sirt7 between RSL1D1 and RAN, which was responsible for the acetylated alteration between RSL1D1 and RAN. Our study indicated that the acetylation of RAN was responsible for the subcellular localization of STAT3. Interestingly, Sirt7 was found to regulate the nuclear export of p65 by deacetylating RAN [28]. Given that the regulation of STAT3 nuclear export is very complex and the nuclear localization of STAT3 usually requires phosphorylation and dimerization [42, 43], further study will be necessary to conclude the protein-protein interactions of RSL1D1/RAN/Sirt7 and the mechanism of acetylated RAN on the subcellular localization of STAT3. Besides STAT3, many key molecules shuttle between nuclear and cytoplasm depending on the effects of RAN [44]. Moreover, it has been suggested p53 may affects the autophagy program [45]. Therefore, wild-type or mutated p53 may affects the autophagy regulated by RSL1D1. Notably, a recent work showed that RSL1D1 regulates p53 in CRC cells [46]. More studies are needed to better understand the role of RSL1D1 in autophagy.

In summary, our study uncovered the key role of the RSL1D1/RAN/STAT3 regulatory axis in autophagy and tumor progression in CRC, highlighting a new target for preventing and treating CRC.

## Materials and methods

### Cell cultures, reagents, and antibodies

The human CRC cell lines, include SW480, SW620, LoVo, DLD1, RKO, and HCT116, NCM460 (Normal human colon mucosal epithelial cell) and HEK293T (Human embryonic kidney cell) were obtained from the Cell Bank at the Chinese Academy of Sciences (Shanghai, People’s Republic of China). All the cell lines were cultured in RPMI-1640 with 10% fetal bovine serum (FBS; Gibco, USA) and 1% antibiotics (M&C Gene Technology, China) in a humidified atmosphere containing 5% CO_2_ at 37 °C. To induce starvation, cells were washed twice with PBS, followed by the addition of Earles Balanced Salt Solution (EBSS) (Fdbio science, FD7061) and cultured for 2 h. The Lentiviral vector LV-RSL1D1-RNAi (Gen Chem, Shanghai, China) containing puromycin resistance gene, luciferase gene, and RSL1D1 silencing gene was transfected into SW480 cells and LoVo cells. Besides, the lentiviral vector LV-RSL1D1 (Gen Chem, Shanghai, China) containing puromycin resistance gene, luciferase gene, 3×Flag gene, and RSL1D1 overexpression gene was transfected into HCT116 cells and DLD1 cells. Then, transduced cells were selected for 14 days with 2.5 ug/mL puromycin (S7417, Selleckchem, USA). Protein and mRNA of transfected cells were extracted for qPCR and WB analyses. Inhibitors, Nicotinamide (NAM, Selleckchem, USA) was used at 10 mM for 6 h; Chloroquine (CQ, C6628, Sigma-Aldrich) was used at 10 μM for 24 h. Rapamycin (RAPA, Selleckchem, USA), was used at 100 nM for 24 h. The following primary antibodies were used: Sirt7 (Rabbit mAb, 5360, CST), STAT3 (Mouse mAb, 9139, CST) and p-STAT3Y705 (Rabbit mAb, 9145, CST), RSL1D1 (Rabbit mAb, ab181100, Abcam), RAN (Rabbit mAb, 0469-1-AP, Proteintech), RAN (Mouse mAb, 271376, Santa Cruz), GAPDH (Mouse mAb, 60004-1-Ig, Proteintech), Pan Acetylation (Rabbit mAb, 66289-1-Ig, Proteintech), LC3B (Rabbit mAb,14600-1-AP, Proteintech), P62 (Rabbit mAb,18420-1-AP, Proteintech), FLAG (Mouse mAb, 66008-3-Ig, Proteintech), H3 (Rabbit mAb, A2348, ABclonal). The siRNAs were used to silence target genes by transfecting with Lipofectamine 3000 (Life Technologies–Invitrogen, USA), according to the manufacturer’s instructions. The sequences of siRNAs are listed in Supplementary Table 2.

### Tissue samples and evaluation

From 2017 to 2019, 52 pairs of fresh CRC tissues and paired normal colorectal tissues from patients with primary CRC were collected, 16 of them were analyzed by WB, and the others were analyzed by RT-PCR. The TMA was generated from formalin-fixed, paraffin-embedded tissues of 231 patients with CRC, which were collected from 2000 to 2005 at NanFang Hospital, none of these patients received any chemotherapy or radiotherapy before the operation. The RSL1D1 staining score was evaluated using two blinded individuals. IHC was scored as the product of tumor cell staining intensity (0–3) and percentage of cells positive (0–100) (overall product score range = 0–300). The median values of the scores (200) were used as cutoff points to classify CRC as ‘RSL1D1 low expression’ or ‘RSL1D1 high expression’.

### Immunofluorescence staining

Cells seeded on coverslips were fixed in methyl alcohol and permeabilized with 0.1% Triton X-100 (Sigma, T9284). Then, cells were blocked with 5% bovine serum albumin (BSA; Sigma, B2064) at room temperature for 1 h and incubated with primary antibody overnight at 4 °C, followed by incubation with Alexa Fluor 488-conjugated goat antirabbit IgG (Abbkine, A23220) and Alexa Fluor 594-conjugated goat antimouse IgG (Abbkine, A23410) at room temperature for 1 h. Nuclei were stained by DAPI (Beyotime, C1006) for 15 min. Finally, images were taken under Karl Zeiss inverted laser confocal microscope (LSM 880 with Airyscan, Germany). Cells expressing mCherry-EGFP-LC3B were treated with DAPI for 15 min after fixation, then the cells were observed with the confocal microscope directly. Autophagy was measured by an observer blinded to experimental condition with quantitation of colored puncta per cell.

### Cell extraction and Western blotting

Cellular proteins were harvested and lysed in RIPA buffer (1% NP-40, 0.25% sodium deoxycholate) containing protease inhibitors (Roche). Equal proteins were electrophoresed and transferred onto PVDF membranes (Merck millpore). Protein expression was detected using primary and secondary antibodies and visualized with an enhanced chemiluminescence system. The specific bands were quantified using Image J (Version: 1.50 G).

### Coimmunoprecipitation (co-IP) and deacetylase assay

Cells were harvested by centrifugation at 4 °C 1400 g for 5 min and washed with cold PBS for three times. Then the cell pellet was resuspended in the pre-cooled RIPA buffer on ice for 20 min and centrifuged at 4 °C 1400 g for 15 min. The supernatant was collected in a new EP tube. Antibody (1 to 2 g) was added to 1 ml of cell lysate and incubated at 4 °C for 12 h. After adding protein A/G agarose beads (Santa Cruz Biotechnology), the incubation was continued for 8 to 12 h at 4 °C. The immunoprecipitants were washed with washing buffer and repeated five times, and eluted with 2×SDS loading buffer by boiling for 5 min. Finally, the samples were separated by SDS-PAGE and analyzed by WB. To detect lysine acetylation of RSL1D1 or RAN, the cell lysates were incubated with RSL1D1 antibody or RAN antibody, then repeated the above steps, the samples were analyzed by WB using an anti-acetylated-lysine antibody in the end.

### Transmission electron microscopy (TEM)

Cells plated at 2 × 10^6^ cells/mL were treated. In a primary case, each sample was fixed with 2% glutaraldehyde at 4 °C overnight and washed three times with 0.1 M phosphate-buffered saline (PBS; Gibco, 10010023). Samples were then postfixed with 1% OsO4 dissolved in 0.1 M PBS for 2 h and dehydrated using an ascending gradual series (50–100%) of ethanol and infiltrated with propylene oxide. After sectioning and staining with uranyl acetate and lead citrate, the samples were viewed via TEM (HITACHI, HT7700, Japan).

### RNA extraction and qPCR analysis

Total RNAs were purified using TRIzol (Invitrogen, Carlsbad, CA). The cDNA was generated with PrimeScript RT kit (Promega, Madison, WI, USA). RT-PCR was carried out using SYBR Premix EX Taq (TaKaRa) on ABI7500 Real-time PCR system (Applied Biosystems, Foster City, USA). The primers for PCR reactions are listed in Supplementary Table 3.

### Cell proliferation assay and Colony formation assay

1 × 10^3^ cells were seeded on 96-well plates and cultured for 24 h. The Cell viability was determined every day by using CCK-8 (Dojindo Laboratories, CK04) according to the manufacturer’s instructions. Two hundred cells per well were inoculated on a 6-well plate and cultured for 2 weeks. Cells were fixed with methyl alcohol for 15 min, and subjected to 1% Giemsa for 15 min. Count the number of colonies with cells more than 50. All observations were reproduced at least three times in independent experiments.

### Wound-healing assays and Matrigel Transwell invasion assay

For wound-healing assays, cells were seeded into 12-well plates. The confluent monolayers were wounded in a line across the plates with a sterile 20 mL plastic pipette tips. All cellular debris was removed by washing with PBS. The area of migration was quantified using Image J. For Matrigel Transwell invasion assay, the upper chamber of the Transwell chamber (BD, USA) was precoated with 200 ug/ml Matrigel (Corning Lot. 9189007), and 2 × 10^5^ cells suspended in serum-free media were placed in the upper compartment of the Transwells and 20% FBS was used as a chemo-attractant filled in the lower compartment. The cells were cultured at 37 °C for 2–3 days. Then, cells were fixed with methanol and stained with Giemsa. To quantify the migratory cells microscopically, cells were counted in five random fields (magnification, 200×). All the experiments were performed in at least triplicate.

### Xenografts

All manipulations involving live mice were provided by the Experimental Animal Center of Southern Medical University and certified by Guangdong Provincial Science Bureau. To evaluate in vivo tumor growth, 2 × 10^6^ cells were subcutaneously injected into nude mice (BALB/c, SPF grade, Female, 4–5 weeks old, least 5 per group). The formula (volume (mm^3^) = width^2^ (mm^2^) × length (mm)/2) was used to calculate the tumor sizes. Tumor sizes were measured every 5 days. Then, 8 weeks later, mice were killed and tumors were surgically resected, dehydrated, fixed, embedded in paraffin, and sectioned. The sections were stained with hematoxylin-eosin and observed under the microscope. To evaluate in vivo tumor metastasis, 2×10^6^ cells were injected into the subserous layer of the cecum in nude mice (BALB/c, SPF grade, Female, 4–5 weeks old, least 5 per group). 45 days later, a scheme of non-invasive detection of luciferase activity in nude mice expressing Luc by bioluminescence (Promega) using an instrument (FX Pro, USA). The mice were then killed on the 60th day after surgery, and the tumors were removed for assessments. Three mutual experimenters are responsible for grouping using a blinding and randomization method, processing, and data collection. All experimental procedures were performed in strict accordance with the recommendations in the Guide for the Care and Use of Laboratory Animals of the National Institutes of Health. The protocol was approved by the Committee on the Ethics of Animal Experiments of Southern Medical University.

### Statistical analysis

Each assay was performed in three independent experiments. Statistical analyses were performed using SPSS software (version 23.0, IBM Corp, Armonk, NY, USA). All data followed a normal distribution with homogenous variance. The independent Student’s *t* test (two-tailed) or one-way analysis of variance (ANOVA). Kaplan–Meier analyses were used for survival analysis. A *P* < 0.05 was considered statistically significant (*P < 0.05, **P < 0.01, ***P < 0.001, NS means no statistic difference). The error bars represent mean ± SD.

## Supplementary information

Supplementary materials

Reproducibility checklist

## Data Availability

The datasets generated and/or analyzed during the current study are not publicly available but are available from the corresponding author on reasonable request.

## References

[1] Siegel RL, Miller KD, Jemal A (2019). Cancer statistics, 2019. CA Cancer J Clin.

[2] Carrato A, Abad A, Massuti B, Gravalos C, Escudero P, Longo-Munoz F (2017). First-line panitumumab plus FOLFOX4 or FOLFIRI in colorectal cancer with multiple or unresectable liver metastases: A randomised, phase II trial (PLANET-TTD). Eur J Cancer.

[3] Corcoran RB, Andre T, Atreya CE, Schellens JHM, Yoshino T, Bendell JC (2018). Combined BRAF, EGFR, and MEK Inhibition in Patients with BRAF(V600E)-Mutant Colorectal Cancer. Cancer Discov.

[4] Le DT, Durham JN, Smith KN, Wang H, Bartlett BR, Aulakh LK (2017). Mismatch repair deficiency predicts response of solid tumors to PD-1 blockade. Science.

[5] Vasaikar S, Huang C, Wang X, Petyuk VA, Savage SR, Wen B (2019). Proteogenomic Analysis of Human Colon Cancer Reveals New Therapeutic Opportunities. Cell.

[6] Hu J, Zhu X, Ye K (2017). Structure and RNA recognition of ribosome assembly factor Utp30. RNA.

[7] Schilling V, Peifer C, Buchhaupt M, Lamberth S, Lioutikov A, Rietschel B (2012). Genetic interactions of yeast NEP1 (EMG1), encoding an essential factor in ribosome biogenesis. Yeast.

[8] Guo S, Zhang Z, Tong T (2004). Cloning and characterization of cellular senescence-associated genes in human fibroblasts by suppression subtractive hybridization. Exp Cell Res.

[9] Herranz N, Gil J (2018). Mechanisms and functions of cellular senescence. J Clin Invest.

[10] Cheng Q, Yuan F, Lu F, Zhang B, Chen T, Chen X (2015). CSIG promotes hepatocellular carcinoma proliferation by activating c-MYC expression. Oncotarget.

[11] Perou CM, Sorlie T, Eisen MB, van de Rijn M, Jeffrey SS, Rees CA (2000). Molecular portraits of human breast tumours. Nature.

[12] Li XP, Jiao JU, Lu LI, Zou Q, Zhu S, Zhang Y (2016). Overexpression of ribosomal L1 domain containing 1 is associated with an aggressive phenotype and a poor prognosis in patients with prostate cancer. Oncol Lett.

[13] Glick D, Barth S, Macleod KF (2010). Autophagy: cellular and molecular mechanisms. J Pathol.

[14] Kroemer G, Marino G, Levine B (2010). Autophagy and the integrated stress response. Mol Cell.

[15] Zhi X, Zhong Q (2015). Autophagy in cancer. F1000Prime Rep.

[16] Rajendran P, Alzahrani AM, Hanieh HN, Kumar SA, Ben Ammar R, Rengarajan T (2019). Autophagy and senescence: A new insight in selected human diseases. J Cell Physiol.

[17] Garcia-Prat L, Martinez-Vicente M, Perdiguero E, Ortet L, Rodriguez-Ubreva J, Rebollo E (2016). Autophagy maintains stemness by preventing senescence. Nature.

[18] Kang C, Elledge SJ (2016). How autophagy both activates and inhibits cellular senescence. Autophagy.

[19] Tang Z, Li C, Kang B, Gao G, Li C, Zhang Z (2017). GEPIA: a web server for cancer and normal gene expression profiling and interactive analyses. Nucleic Acids Res.

[20] Ma L, Chang N, Guo S, Li Q, Zhang Z, Wang W (2008). CSIG inhibits PTEN translation in replicative senescence. Mol Cell Biol.

[21] Goldman MJ, Craft B, Hastie M, Repecka K, McDade F, Kamath A (2020). Visualizing and interpreting cancer genomics data via the Xena platform. Nat Biotechnol.

[22] Klionsky DJ, Abdel-Aziz AK, Abdelfatah S, Abdellatif M, Abdoli A, Abel S (2021). Guidelines for the use and interpretation of assays for monitoring autophagy (4th edition)(1). Autophagy.

[23] Fullgrabe J, Klionsky DJ, Joseph B (2014). The return of the nucleus: transcriptional and epigenetic control of autophagy. Nat Rev Mol Cell Biol.

[24] Liu L, McBride KM, Reich NC (2005). STAT3 nuclear import is independent of tyrosine phosphorylation and mediated by importin-alpha3. Proc Natl Acad Sci USA.

[25] You L, Wang Z, Li H, Shou J, Jing Z, Xie J (2015). The role of STAT3 in autophagy. Autophagy.

[26] Sazer S, Dasso M (2000). The ran decathlon: multiple roles of Ran. J Cell Sci.

[27] de Boor S, Knyphausen P, Kuhlmann N, Wroblowski S, Brenig J, Scislowski L (2015). Small GTP-binding protein Ran is regulated by posttranslational lysine acetylation. Proc Natl Acad Sci USA.

[28] Sobuz SU, Sato Y, Yoshizawa T, Karim F, Ono K, Sawa T (2019). SIRT7 regulates the nuclear export of NF-kappaB p65 by deacetylating Ran. Biochim Biophys Acta Mol Cell Res.

[29] Lee N, Kim DK, Kim ES, Park SJ, Kwon JH, Shin J (2014). Comparative interactomes of SIRT6 and SIRT7: Implication of functional links to aging. Proteomics.

[30] Calcinotto A, Kohli J, Zagato E, Pellegrini L, Demaria M, Alimonti A (2019). Cellular Senescence: Aging, Cancer, and Injury. Physiol Rev.

[31] Yuan F, Zhang Y, Ma L, Cheng Q, Li G, Tong T (2017). Enhanced NOLC1 promotes cell senescence and represses hepatocellular carcinoma cell proliferation by disturbing the organization of nucleolus. Aging Cell.

[32] Liang XH, Jackson S, Seaman M, Brown K, Kempkes B, Hibshoosh H (1999). Induction of autophagy and inhibition of tumorigenesis by beclin 1. Nature.

[33] Takamura A, Komatsu M, Hara T, Sakamoto A, Kishi C, Waguri S (2011). Autophagy-deficient mice develop multiple liver tumors. Genes Dev.

[34] Boya P, Reggiori F, Codogno P (2013). Emerging regulation and functions of autophagy. Nat Cell Biol.

[35] Guo JY, Karsli-Uzunbas G, Mathew R, Aisner SC, Kamphorst JJ, Strohecker AM (2013). Autophagy suppresses progression of K-ras-induced lung tumors to oncocytomas and maintains lipid homeostasis. Genes Dev.

[36] Moore MS, Blobel G (1993). The GTP-binding protein Ran/TC4 is required for protein import into the nucleus. Nature.

[37] Scheffzek K, Klebe C, Fritz-Wolf K, Kabsch W, Wittinghofer A (1995). Crystal structure of the nuclear Ras-related protein Ran in its GDP-bound form. Nature.

[38] Melchior F, Paschal B, Evans J, Gerace L (1993). Inhibition of nuclear protein import by nonhydrolyzable analogues of GTP and identification of the small GTPase Ran/TC4 as an essential transport factor. J Cell Biol.

[39] Knyphausen P, Kuhlmann N, de Boor S, Lammers M (2015). Lysine-acetylation as a fundamental regulator of Ran function: Implications for signaling of proteins of the Ras-superfamily. Small GTPases.

[40] Blank MF, Grummt I (2017). The seven faces of SIRT7. Transcription.

[41] Kiran S, Chatterjee N, Singh S, Kaul SC, Wadhwa R, Ramakrishna G (2013). Intracellular distribution of human SIRT7 and mapping of the nuclear/nucleolar localization signal. FEBS J.

[42] Bhattacharya S, Schindler C (2003). Regulation of Stat3 nuclear export. J Clin Invest.

[43] Levy DE, Darnell JE (2002). Stats: transcriptional control and biological impact. Nat Rev Mol Cell Biol.

[44] Lui K, Huang Y (2009). RanGTPase: A Key Regulator of Nucleocytoplasmic Trafficking. Mol Cell Pharm.

[45] Rosenfeldt MT, O’Prey J, Morton JP, Nixon C, MacKay G, Mrowinska A (2013). p53 status determines the role of autophagy in pancreatic tumour development. Nature.

[46] Ding L, Zhang Z, Zhao C, Chen L, Chen Z, Zhang J (2021). Ribosomal L1 domain-containing protein 1 coordinates with HDM2 to negat ely regulate p53 in human colorectal Cancer cells. J Exp Clin Cancer Res.

