# Supplementary material for: RSL1D1 promotes the progression of colorectal cancer through RAN-mediated autophagy suppression
Source: Cell Death Dis. 2022 Jan 10;13(1):43. doi: 10.1038/s41419-021-04492-z (PMC8748816; doi:10.1038/s41419-021-04492-z)

- **Supplementary Table-1** ***The Relationship between RSL1D1 expression and CRC clinicopathological features***

**Characteristics**	**N=231(%)**	**IHC Score of RSL1D1 mean ± SD**	**F**	**P**
**Gender**			0.025	**0.87**
Male	136(59%)	203.38±57.19		
Female	95(41%)	204.58±55.72		
**Age (years)**			1.32	**0.25**
< 50	61(26%)	210.98±55.22		
≥50	170(74%)	201.32±56.47		
**Tumor size (cm)**			0.09	**0.77**
< 5	132(57.1%)	202.92±52.96		
≥5	99(42.9%)	205.15±61.09		
**Differentiation**			8.55	**<0.001**
Well	72(31.2%)	184.79±56.79		
Moderate	102(44.2%)	205.74±52.40		
Poor	57(24.7%)	224.65±56.06		
**T stage**			2.676	**0.048**
T1	1(4%)	180±0		
T2	32(13%)	191.56±63.28		
T3	185(79.9%)	203.41±53.85		
T4	13(6.7%)	242.69±64.96		
**N classification**			8.1	**0.005**
N0	80(34.63%)	189.56±53.15		
N1-2	151(67.8%)	211.46±56.87		
**M classification**			4.9	**0.028**
M0	187(81.00%)	199.92±55.74		
M1	44(19.0%)	220.68±55.09		
**Clinical stage**			8.26	**0.004**
I+II	70(30.3%)	187.93±52.03		
III+IV	161(69.7%)	210.81±57.07		

- **Supplementary Table-2** ***The sequences of siRNAs used in our experiments***

Gene	Sequence
**CTRL**	TTCTCCGAACGTGTCACGT
**RSL1D1**	siRSL1D1-1: GCCTCATAGTATTCGATCA<br>siRSL1D1-2: GTGGAACAGTCTTAAACAT<br>siRSL1D1-3: CTGCATCAGTTCTTAGTAA
**RAN**	siRAN-1: ACAGTATGAGCACGACTTA<br>siRAN-2: CCCTAACTTGGAATTTGTT<br>siRAN-3: GGATATTAAGGACAGGAAA
**Sirt7**	siSirt7-1: AGCCATTTGTCCTTGAGGAA<br>siSirt7-2: GAACGGAACTCGGGTTATT

- **Supplementary Table-3** ***The sequences of primers used in our experiments.***

Gene		Primer sequences 5'—— 3'
**RSL1D1**	F	TGCTATACGTATTGGTCACGTT
	R	TTTCACAAACAGGAGTTTCACG
**GAPDH**	F	ACAGTCAGCCGCATCTTCTT
	R	GACAAGCTTCCCGTTCTCAG
**RAN**	F	GAAACGTCATTTGACTGGTGAA
	R	ACTGGGCTTGGATATAATAGCC
**ATG5**	F	GATGGGATTGCAAAATGACAGA
	R	GAAAGGTCTTTCAGTCGTTGTC
**ULK1**	F	CTCCTTTGACTTCCCGAAGAC
	R	CTTAAGGAGCAGGTCAGTGAG
**BECN1**	F	GAAACGTCATTTGACTGGTGAA
	R	ACTGGGCTTGGATATAATAGCC
**LC3B**	F	ACTGTCTCGCGTAGTAGGTG
	R	GGTCACCCCTCAGCATGTTA
**BNIP3**	F	AGGGCTCCTGGGTAGAACT
	R	CTCCATTATAAATAGAAACCGAGGC
**ATG3**	F	CGGTGCAAACAGATGGAATATT
	R	GTGTGATCTCTTTAACGGCTTC
**BCL2**	F	GACTTCGCCGAGATGTCCAG
	R	GAACTCAAAGAAGGCCACAATC

Supplementary Figure legends

- **Supplementary Fig-1 A.** Establishment of CRC cells stably overexpressing RSL1D1. **B.** Establishment of CRC cells with stable silencing of RSL1D1. **C.** Efficiency detection of RNAi silencing of RAN. *P < 0.05, **P < 0.01, ***P < 0.001, NS means no statistic difference. The error bars represent mean ± SD.

- **Supplementary Fig-2 A.** GO analysis of RSL1D1 in CRC data from TCGA. **B.** Protein expression of P62 and LC3B examined by WB after RSL1D1 overexpression or knockdown in CRC cells under normal conditions (10% FBS). **C.** CRC cells with RSL1D1 downregulation and overexpression were transfected with mCherry-EGFP-LC3B, and the changes in green and red fluorescence were observed using a confocal microscope under normal conditions. Scale bar, 20 μm. **D.** Protein expression of P62 and LC3B examined by WB in CRC cells after RSL1D1 knockdown and/or treatment with CQ. **E.** Protein expression of P62 and LC3B examined by WB in CRC cells after RSL1D1 overexpression and/or treatment with RAPA. *P < 0.05, **P < 0.01, ***P < 0.001, NS means no statistic difference. The error bars represent mean ± SD.

- **Supplementary Fig-3 A.** mRNA levels of STAT3-regulated genes in the process of autophagy under starvation conditions. **B.** Co-IP of endogenous RSL1D1 and STAT3 proteins in CRC cells.

- **Supplementary Fig-4 A.** Co-IP of endogenous RAN and Sirt7 proteins in CRC cells. **B.** Double IF staining revealed the colocalization of RAN and Sirt7 proteins in CRC cells. **C.** Co-IP of endogenous RSL1D1 and Sirt7 proteins in CRC cells and 293T cells. Scale bar, 20 μm. **D.** Double IF staining revealed the colocalization of RSL1D1

and Sirt7 proteins in CRC cells and 293T cells. Scale bar, 50 μm. **E.** Acetylation of endogenous RAN in CRC cells treated with the deacetylase inhibitor NAM or transfected with siRNA-Sirt7. **F.** Acetylation of endogenous or exogenous RSL1D1 in CRC cells treated with the deacetylase inhibitor NAM or transfected with siRNA-Sirt7. **G.** Acetylation of RAN in CRC cells after RSL1D1 knockdown or overexpression.

Supplementary Figure-1

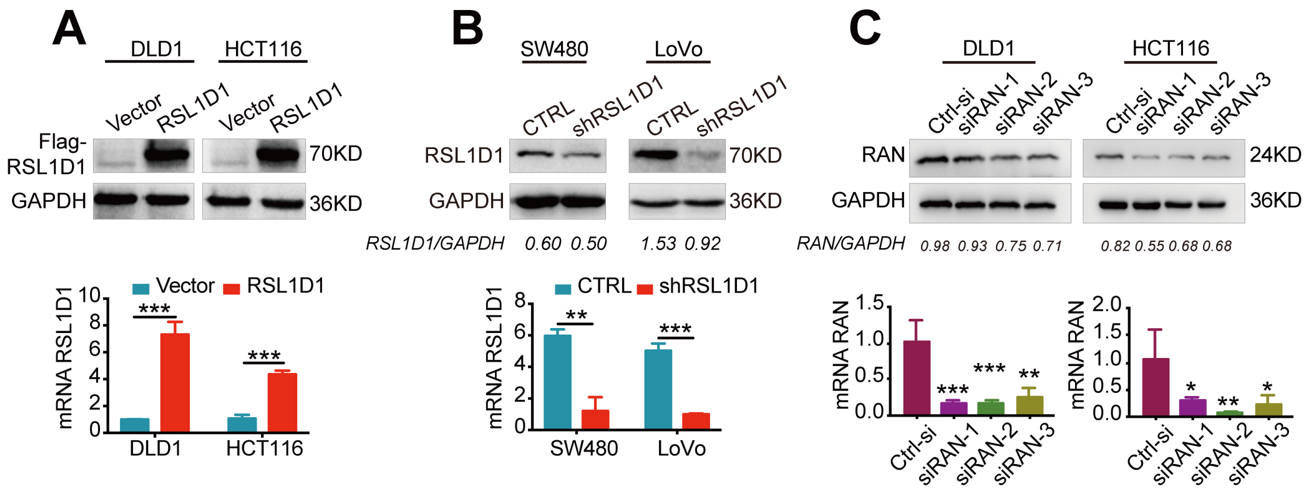

Supplementary Figure-2

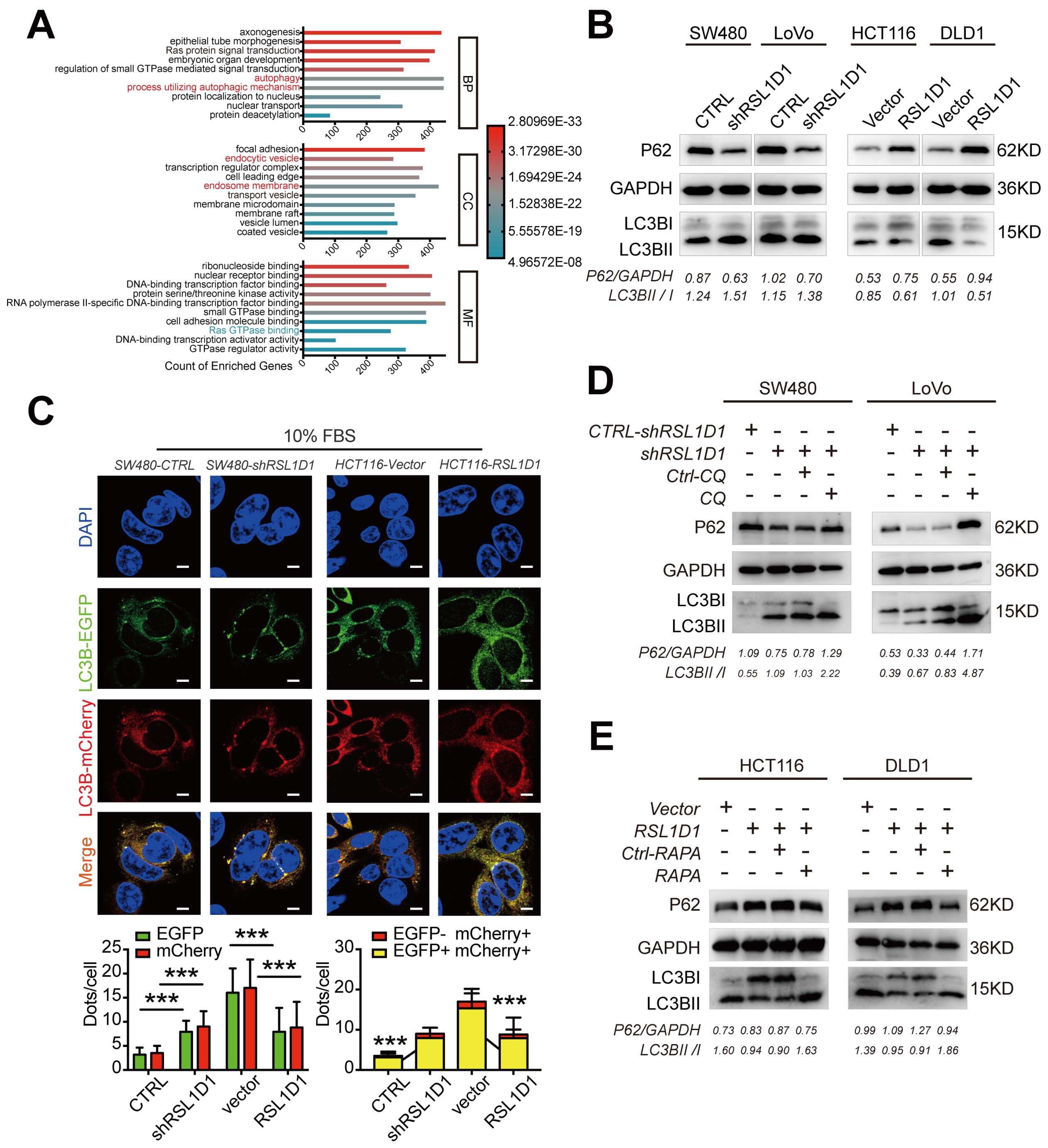

Supplementary Figure-3

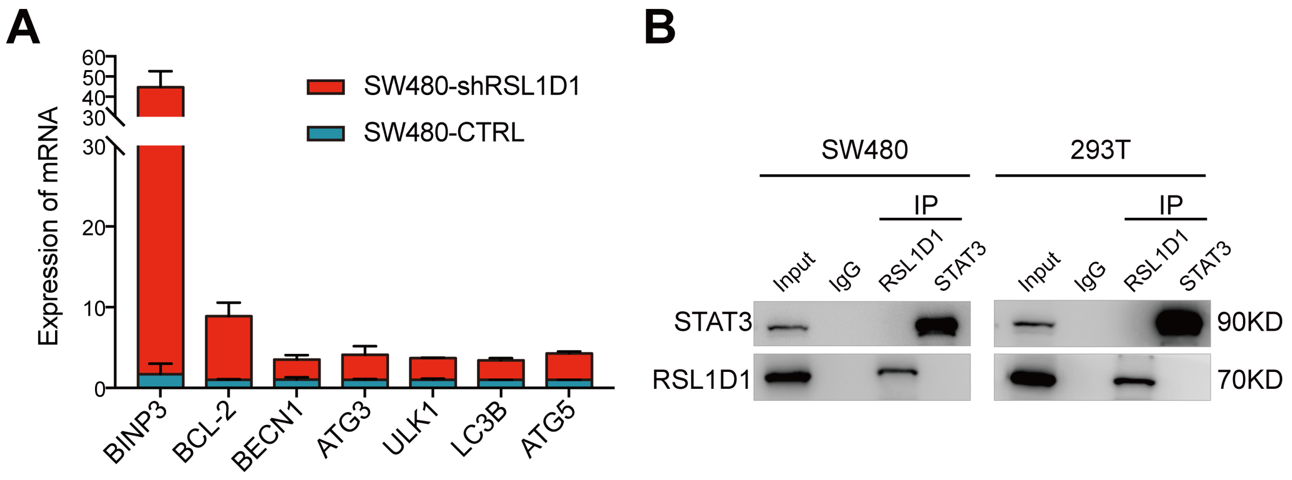

Supplementary Figure-4

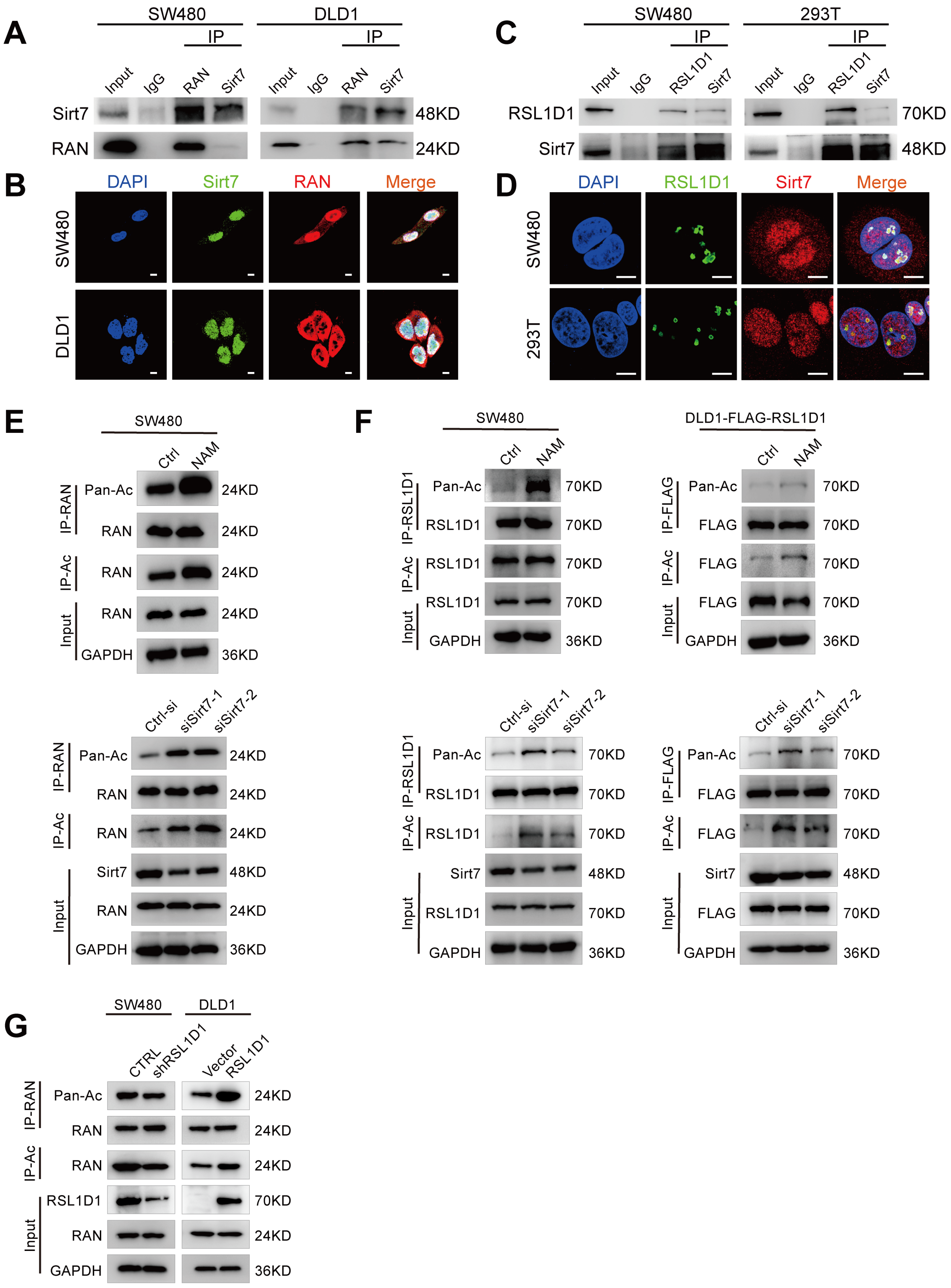

Supplement: Supplementary file 1 — Supplementary materials [file 41419_2021_4492_MOESM1_ESM.pdf]
